# Effect of participatory women's groups and counselling through home visits on children's linear growth in rural eastern India (CARING trial): a cluster-randomised controlled trial

**DOI:** 10.1016/S2214-109X(17)30339-X

**Published:** 2017-09-11

**Authors:** Nirmala Nair, Prasanta Tripathy, H S Sachdev, Hemanta Pradhan, Sanghita Bhattacharyya, Rajkumar Gope, Sumitra Gagrai, Shibanand Rath, Suchitra Rath, Rajesh Sinha, Swati Sarbani Roy, Suhas Shewale, Vijay Singh, Aradhana Srivastava, Anthony Costello, Andrew Copas, Jolene Skordis-Worrall, Hassan Haghparast-Bidgoli, Naomi Saville, Audrey Prost

**Affiliations:** a Ekjut, Chakradharpur, Jharkhand, India; b Sitaram Bhartia Institute of Science and Research, New Delhi, India; c Public Health Foundation of India, New Delhi, India; d Institute for Global Health, University College London, London, UK

## Abstract

**Background:**

Around 30% of the world's stunted children live in India. The Government of India has proposed a new cadre of community-based workers to improve nutrition in 200 districts. We aimed to find out the effect of such a worker carrying out home visits and participatory group meetings on children's linear growth.

**Methods:**

We did a cluster-randomised controlled trial in two adjoining districts of Jharkhand and Odisha, India. 120 clusters (around 1000 people each) were randomly allocated to intervention or control using a lottery. Randomisation took place in July, 2013, and was stratified by district and number of hamlets per cluster (0, 1–2, or ≥3), resulting in six strata. In each intervention cluster, a worker carried out one home visit in the third trimester of pregnancy, monthly visits to children younger than 2 years to support feeding, hygiene, care, and stimulation, as well as monthly women's group meetings to promote individual and community action for nutrition. Participants were pregnant women identified and recruited in the study clusters and their children. We excluded stillbirths and neonatal deaths, infants whose mothers died, those with congenital abnormalities, multiple births, and mother and infant pairs who migrated out of the study area permanently during the trial period. Data collectors visited each woman in pregnancy, within 72 h of her baby's birth, and at 3, 6, 9, 12, and 18 months after birth. The primary outcome was children's length-for-age *Z* score at 18 months of age. Analyses were by intention to treat. Due to the nature of the intervention, participants and the intervention team were not masked to allocation. Data collectors and the data manager were masked to allocation. The trial is registered as ISCRTN (51505201) and with the Clinical Trials Registry of India (number 2014/06/004664).

**Results:**

Between Oct 1, 2013, and Dec 31, 2015, we recruited 5781 pregnant women. 3001 infants were born to pregnant women recruited between Oct 1, 2013, and Feb 10, 2015, and were therefore eligible for follow-up (1460 assigned to intervention; 1541 assigned to control). Three groups of children could not be included in the final analysis: 147 migrated out of the study area (67 in intervention clusters; 80 in control clusters), 77 died after the neonatal period and before 18 months (31 in intervention clusters; 46 in control clusters), and seven had implausible length-for-age *Z* scores (<–5 SD; one in intervention cluster; six in control clusters). We measured 1253 (92%) of 1362 eligible children at 18 months in intervention clusters, and 1308 (92%) of 1415 eligible children in control clusters. Mean length-for-age *Z* score at 18 months was −2·31 (SD 1·12) in intervention clusters and −2·40 (SD 1·10) in control clusters (adjusted difference 0·107, 95% CI −0·011 to 0·226, p=0·08). The intervention did not significantly affect exclusive breastfeeding, timely introduction of complementary foods, morbidity, appropriate home care or care-seeking during childhood illnesses. In intervention clusters, more pregnant women and children attained minimum dietary diversity (adjusted odds ratio [aOR] for women 1·39, 95% CI 1·03–1·90; for children 1·47, 1·07–2·02), more mothers washed their hands before feeding children (5·23, 2·61–10·5), fewer children were underweight at 18 months (0·81, 0·66–0·99), and fewer infants died (0·63, 0·39–1·00).

**Interpretation:**

Introduction of a new worker in areas with a high burden of undernutrition in rural eastern India did not significantly increase children's length. However, certain secondary outcomes such as self-reported dietary diversity and handwashing, as well as infant survival were improved. The interventions tested in this trial can be further optimised for use at scale, but substantial improvements in growth will require investment in nutrition-sensitive interventions, including clean water, sanitation, family planning, girls' education, and social safety nets.

**Funding:**

UK Medical Research Council, Wellcome Trust, UK Department for International Development (DFID).

**Research in context**

**Evidence before this study**

Systematic reviews have examined the independent effects of breastfeeding promotion, complementary feeding education, prenatal and postnatal food and micronutrient supplementation, psychosocial stimulation, as well as water, sanitation, and hygiene interventions on height-for-age *Z* score or length-for-age *Z* score among children younger than 5 years in low-income and middle-income countries. All found either small (<0·25 SD) or null effects on height-for-age *Z* score or length-for-age *Z* score, leading to calls for combining some or all of these interventions, and an increased focus on nutrition-sensitive interventions.

We did a systematic review of studies testing the effects of interventions integrating combinations of infant and young child feeding promotion, hygiene through handwashing with soap, prevention and care-seeking for childhood illnesses, and psychosocial stimulation on height-for-age *Z* score or length-for-age *Z* score among children younger than 2 years in South Asia. We did the review before the start of the intervention in 2013, and updated it on March 6, 2017. We searched for randomised controlled trials published between Oct 1, 2000, and March 6, 2017, in English in Embase, MEDLINE, and PsycINFO using the terms “growth”, “stunting”, “height-for-age”, and “length-for-age” in the abstract field. We excluded studies with only severely acutely malnourished children, very low birthweight infants, exclusively formula-fed infants, and those that only measured the length of infants younger than 6 months. Our search yielded 2775 records, from which we identified six relevant trials of moderate-to-good quality from south Asia. In the appendix, we describe these trials and offer a risk assessment. All trials tested the effects of education and coaching on infant and young child feeding practices, including responsive feeding and handwashing. Four trials tested the effect of combining psychosocial stimulation with nutrition education and two trials combined nutrition education and stimulation with multiple micronutrient powder supplementation. All six trials showed either null or small effects on height-for-age *Z* score, length-for-age *Z* score, or attained length, suggesting that combinations of nutrition-specific, stimulation and hygiene promotion interventions are unlikely to lead to substantial gains in linear growth.

**Added value of this study**

To our knowledge, our study was the first to test an integrated community strategy to promote maternal nutrition, infant and young child feeding, hygiene, prevention and care-seeking for childhood illnesses, and stimulation in rural India. The 0·11 SD effect of the intervention on length-for-age *Z* score (an estimated 0·25cm gain in length) was greater than that found in other trials of combined interventions, but still small and non-significant at the 0·05 level.

**Implications of all the available evidence**

Scaling up of nutrition-specific actions, hygiene promotion and responsive stimulation in high-burden settings is crucial to improving children's growth and development. To our knowledge, our combined intervention is the only to date to test the effect of introducing a worker proposed by the Indian Government for rural districts with a high burden of undernutrition, and found effects on self-reported dietary diversity, handwashing, and infant mortality. Further operational research should focus on optimising materials developed in this and other studies for use with accredited social health activists as part of the Mother's Absolute Affection programme and the scale up of participatory learning and action with women's groups in ten states. Achieving the 40% reduction in stunting envisioned by WHO's Global Strategy for Women's, Children's, and Adolescents' health will require investment in nutrition-sensitive interventions including clean water, sanitation, family planning, girls' education, and social safety nets.

## Introduction

An estimated 23% of the world's children are stunted (too short for their age) because of chronic undernutrition.^1^ Growth faltering, which leads to stunting, is fastest between conception and 2 years, or the first 1000 days of life.^2^ Stunting endangers subsequent physical and cognitive development, is linked to poor health and low earnings in adulthood, and contributes to the intergenerational transmission of poverty.^3^ Interventions to promote children's growth have the potential to increase survival and school attainment, offer protection against adult chronic disease, and bolster human development.^3^ WHO's Global Strategy for Women's, Children's and Adolescents' Health and the second Sustainable Development Goal call for urgent action to reduce the number of children who are stunted by 40% by 2025.^4^

There is substantial agreement about the determinants of stunting and interventions to address it, but insufficient operational research for strategies to increase the coverage of these interventions in high-burden settings. *The Lancet*'s 2013 Maternal and Child Nutrition Series recommends ten nutrition-specific interventions, including sufficient and diverse foods for adolescent girls and women; iron, folic acid, and calcium supplementation in pregnancy; good infant and young child feeding practices; vitamin A, iron, and preventive zinc supplementation for children; and treatment for moderate and severe acute malnutrition.^5^ Strikingly however, expanding the coverage of these nutrition-specific interventions to 90% of mothers and children would only reduce stunting by an estimated 20%.^5^ Larger reductions than these require increasing access to family planning, health services for pregnant women and children, investments in water and sanitation, social safety nets, girls' education, and women's empowerment.^6^ A pragmatic approach to design community strategies for stunting reduction might therefore be to prioritise action on immediate determinants (infection control, nutrition, and care in the first 1000 days) while providing an enabling social environment for changes in more distal determinants, for example by sharing information about health, nutrition, and sanitation entitlements, and bolstering women's agency.

India is home to around 30% of the world's stunted children.^7^ 38% of Indian children are too short for their age.^8^ The Government's Integrated Child Development Services (ICDS) and National Health Mission seek to increase the coverage of interventions to promote growth in the first 1000 days of life but face substantial barriers. ICDS' main cadre, the Anganwadi worker, has a workload described by a 2011 Government of India report as “humanly impossible”.^9^ She is asked to provide food supplementation to pregnant women and children aged 6 months to 6 years, monitor the growth of all children younger than 5 years monthly, refer undernourished women and children to health and nutrition services, provide health and nutrition education, support immunisations, and offer preschool education to children aged 3–6 years in a catchment area of 1000 population, all within a 4–5 h working day and for INR3000 (US$44) a month.^9^ India's 12th National Plan (2012–17) proposed a second Anganwadi worker to promote growth among children younger than 3 years in 200 high-burden districts, but no evidence-based, rigorously tested intervention model for such a worker currently exists.^10^ Previous trials^11^, ^12^, ^13^, ^14^, ^15^, ^16^, ^17^, ^18^, ^19^, ^20^, ^21^, ^22^, ^23^ of community interventions to reduce undernutrition have either been done outside India, tested single health, nutrition, or hygiene interventions rather than integrated strategies, did not focus on the 1000 days period, or did not look at the effects on children's length.

We aimed to determine whether a strategy involving a new worker doing home visits and participatory women's group meetings could improve maternal nutrition, as well as feeding, hygiene, care, and stimulation practices for children, and through this, lead to an increase in children's linear growth.

## Methods

### Study design and setting

We did a cluster-randomised controlled trial in West Singhbhum and Kendujhar, two adjoining rural districts of Jharkhand and Odisha in eastern India. 48% of children younger than 5 years in rural Jharkhand are stunted, and 35% in rural Odisha.^8^ More than 80% of families in West Singhbhum and Kendujhar live in rural areas, and around 40% are from indigenous (*adivasi*) communities. Less than 50% of women are literate, and less than 10% of households have access to a toilet facility.^24^

Within each of the two districts, we identified 120 geographical clusters with a population of around 1000 people each to approximate the catchment area of an Anganwadi worker. The trial area covered an estimated total population of 121 531 people. Each cluster included a village and any adjoining hamlets, and was separated from others by natural boundaries (eg, rivers, hills) or distance. We sought written consent from village leaders for the participation of clusters before randomisation. Intervention and control clusters had not been previously exposed to community interventions with participatory groups or home visits, including those tested in previous studies.^25^

### Participants

Individual participants were pregnant women identified and recruited in the study clusters and their children. Community-based, incentivised volunteers identified women in the third trimester of pregnancy. 30 female data collectors (one per 4000 population) with 10–12 years of education verified these identifications, approached each pregnant woman to explain the study, and sought written consent for participation. Data collectors then visited each woman in pregnancy, within 72 h of her baby's birth, and at 3, 6, 9, 12, and 18 months after birth. We continued to identify pregnant women until Dec 31, 2015, to measure the effect of the intervention on maternal nutrition in pregnancy, although we did not follow up these mothers' children. We sought women's individual informed consent in writing or by thumbprint during the enrolment interview in pregnancy, and verbally before all subsequent interviews.

We excluded stillbirths and neonatal deaths, infants whose mothers died, those with congenital abnormalities, multiple births, and mother and infant pairs who migrated out of the study area permanently during the trial period. Permanent migrants were defined as those missing two consecutive interviews and the final interview at 18 months, or missing interviews at 12 and 18 months. Migration was assumed to have occurred at the point of the earliest missed consecutive interview. Data collectors attempted to find each mother at least three times at each follow-up.

Ekjut, a civil society organisation working to improve health in rural indigenous communities of eastern India since 2002, led the intervention and data collection. The trial protocol was reviewed and approved by the research ethics committee of the Public Health Foundation of India (June, 2013, TRC-IEC-163/13), an Independent Ethics Committee linked to Ekjut (May, 2013, reference IEC/EKJUT/01), and University College London's Research Ethics Committee (June, 2013, reference 1881/002). The study was overseen by a trial steering committee of three members and by a data monitoring committee of five members with expertise in paediatrics, nutritional epidemiology, and statistics. Both committees met annually. For ethical reasons, severely acutely malnourished children identified by data collectors in both trial arms were referred to local malnutrition treatment centres. When parents refused to take children to the local malnutrition treatment centre, local village health sanitation and nutrition committee members were requested to follow up and ensure that they were admitted to local malnutrition treatment centres.

### Procedures

Data collectors measured children's length using Shorr boards (Olney, MD, USA), their weight using Tanita BD-590 scales (Mumbai, India), and their mid-upper arm circumference using tapes from UNICEF. They also measured mothers' height using Seca 213 stadiometers, their weight with Seca 874 scales, and mid-upper arm circumference using Seca 212 tapes. Data collectors received 9 days of training on anthropometry and questionnaire administration, and had fortnightly review meetings. We did two anthropometry standardisation exercises with 2660 children aged 6–24 months before the start of data collection, and another 12 months into data collection. We calculated technical error of measurement (TEM) and coefficients of reliability (R) for length, weight, and mid-upper arm circumference.^26^ R for length was 0·98 before data collection and 0·99 1 year later. Data collectors used smartphones to collect data. Supervisors observed 11% of measurements and interviews. We documented dietary practices using 24-h recall through interviews with all mothers or caregivers, and classified food groups using the most recent Food and Agriculture Organization guidelines for the measurement of women's and children's dietary diversity.^27^ Hygiene practices, including handwashing with soap, were assessed using self-reports. The data manager checked the number of interviews completed every week to identify and address possible problems, and downloaded data every 2 weeks to check for errors using automated Do-files and Stata 13 software.

### Randomisation and masking

Randomisation took place in July, 2013, and was stratified by district and number of hamlets per cluster (0, 1–2, or ≥3), resulting in six strata. For transparency, we invited village leaders, front-line health workers and members of local governance bodies to participate in a randomisation meeting. Meeting participants put numbered balls corresponding to clusters in each stratum in a local tombola (lottery device), then sequentially allocated each ball (cluster) to the intervention or control arms. Due to the nature of the intervention, participants and the intervention team were not masked to allocation. The data collection team and data manager were masked to allocation. Intervention and data collection teams met on different days, and had different team leaders.

### Intervention

In the intervention arm, we recruited 60 new female community-based workers called Su-Poshan Karyakarta (SPK), meaning good nutrition worker, in consultation with local village health sanitation and nutrition committees and existing Anganwadi workers. Each SPK worked in her own village and any nearby hamlets, and covered around 1000 people. She had a minimum of 10 years' schooling, was married, preferably from a tribal community, and was paid a monthly stipend of INR3000, equivalent to that of existing Anganwadi workers at the time of the study. Six supervisors recruited by the study team supported ten SPKs each, mirroring the ICDS supervision structure. The SPK's programme of work was designed not to have overlap with the Anganwadi and accredited social health activists' tasks. SPKs and supervisors received 14 days of training during the intervention period, and attended supervision meetings twice a month.

**Figure 1:**
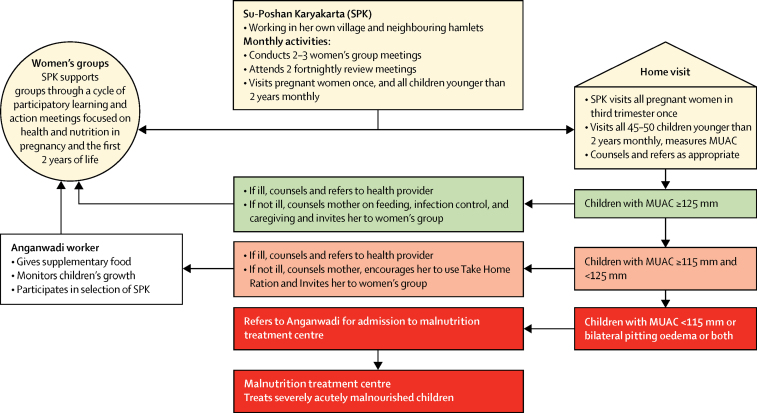
A village-level view of the community intervention strategy MUAC=mid-upper arm circumference.

The SPK was responsible for two main activities: conducting a single home visit to each pregnant woman in the third trimester of pregnancy for counselling on maternal nutrition, followed by monthly home visits to all children younger than 2 years with counselling for growth promotion; and the facilitation of two to three participatory meetings with local women's groups per month (the exact number depended on the size of her working area and number of hamlets; figure 1). Home visits sought to address immediate causes of undernutrition through counselling for infant and young child feeding practices, illness prevention, and support for referrals in case of illness or acute malnutrition. Participatory group meetings reinforced actions linked to immediate causes and began to address underlying causes of undernutrition, including birth spacing, nutrition in pregnancy, water, sanitation, and women's agency.

Before home visits, the SPK gathered the names and location of all pregnant women and children younger than 2 years in her working area from the local Anganwadi worker and members of pre-existing women's groups. At each visit to a mother and child pair, she asked about current or recent illness, took a mid-upper arm circumference measurement for children older than 6 months, and engaged the mother in a discussion about feeding, hygiene, care (defined here as care-seeking for illnesses and offering more food or breastmilk during and after illness), and stimulation. This began with the mothers' immediate concerns with illness, feeding, and care. The SPK used age-appropriate picture cards that depicted good practices and contained key recommendations adapted from the Essential Nutrition Action counselling materials and the Care for Development WHO module about stimulation for early childhood development. She provided information about preventive and care-seeking practices, encouraged the mother to try new practices (eg, new complementary food recipes), demonstrated practices (eg, handwashing, food enrichment, and stimulation), and sought to persuade mothers and other caregivers to adopt them (appendix p 2). Mid-upper arm circumference measurement was used to guide referrals (figure 1).

The SPKs also facilitated a cycle of 29 participatory meetings with women's groups. These monthly meetings targeted pregnant women and mothers of children younger than 2 years and adolescent girls, but were open to all community members. The groups followed a four-phase participatory learning and action cycle in which they: assessed the health and nutrition situation in their community; decided on actions to take; took action; and evaluated the process (appendix p 5). In the first phase, the SPK described the intergenerational cycle of undernutrition using pictures. She encouraged group participants to discuss local practices associated with undernutrition using a picture card game to identify health, nutrition, and care-related problems. Group participants were then invited to prioritise the problems they wanted to address by voting using the picture cards. In the second phase of the cycle, groups explored the causes of their prioritised problems by listening to stories created by the SPKs using local themes. Stories illustrated how problems were linked with underlying household, community, and health service-related factors. Participants decided which of these underlying causes to address, and how. They allocated responsibilities for each strategy and planned a community meeting to share their plans with others and enlist their support. In the third phase, group participants implemented their strategies, and the SPK introduced them to further practical activities, including how to enhance the density and diversity of complementary foods using local products and toy-making for children. In the fourth phase, group participants reviewed their achievements, difficulties, and evaluated individual meetings. Village health sanitation and nutrition committees are composed of elected members of the Panchayat (local governance body), front-line health and nutrition workers, service users (especially mothers), and members from key community subgroups. The committee's role is to monitor access to essential public services, organise local collective action for health promotion, facilitate service delivery in the village and hamlets, make a village health plan, and monitor the quality of local health facilities. In both intervention and control clusters, Ekjut coordinators held five participatory meetings with village health sanitation and nutrition committees in between the committees' regular monthly meetings for 2 years as a minimum common benefit to villages in both trial arms. Meetings aimed to strengthen the capacity of village health sanitation and nutrition committees to assess community health needs, prepare and implement village health plans, and monitor the provision of local health and nutrition services. This was the only activity implemented in control clusters besides routine government services.

### Outcomes

The trial's primary outcome was children's mean length-for-age Z scores at 18 months. Secondary outcomes included wasting and underweight at all timepoints, birthweight, growth velocity, feeding, hygiene, and care practices. Outcomes were published in the trial protocol.^28^ The number of secondary outcomes was later reduced in the online trial registration form after feedback from the data monitoring committee (DMC) in September, 2015.

### Statistical methods

We estimated that the intervention would lead to a 0·15 difference in length-for-age *Z* score at 18 months, based on a meta-analysis of nutrition education interventions that found a mean effect size of 0·2 (range 0·04–0·64) for length-for-age *Z* score at 18 months.^29^ We estimated the intra-cluster correlation for stunting at 0·035 using an analysis of 42 Demographic and Health Surveys.^30^ With around 25 livebirths per year in each cluster of 1000 people and accounting for 10% attrition, recruiting all eligible children born over 12 months in 110 clusters (2520 after attrition) would allow us to detect a difference of 0·15 in mean length-for-age *Z* scores between intervention and control arms, with 80% power and 5% significance level.

We did analyses by intention to treat and conducted in accordance with our DMC-approved data analysis plan. We calculated mean differences for continuous outcomes and odds ratios for binary outcomes. We included random effects for clusters in regression models. We assessed the normality of data and variance for the primary outcome in each study arm. Based on a two-way scatter plot, we found no evidence that the mean or variance of the residuals from the full model were associated with the predicted value of the outcome, and therefore considered linear regression appropriate. We considered the adjusted effect measures as the main results. On the basis of a priori opinion concerning predictors of the primary outcome, we adjusted all child outcomes for sex, district, multidimensional poverty index category, residence (village or hamlet), and tribal status (tribal or not). For anthropometry outcomes, we planned to test differences between arms at earlier timepoints only if a significant difference was seen at 18 months. For child dietary diversity and meal frequency, we calculated summary effect measures across 12 months and 18 months by including an additional random effect for child and adjusting for timepoint. Following our analysis plan, we investigated the effect of intervention intensity by examining effect measures for intensity subgroups relative to the control arm. However, testing of the effect of intensity was based on analysis in the intervention arm only. To investigate whether the intervention effect varied by either infant sex or multidimensional poverty index we present effect measures by subgroup and test the interaction between subgroup and the intervention. We did subgroup analyses for intervention intensity and multidimensional poverty, treating these factors as continuous or categorical so as to detect associations that might or might not be roughly linear.

A priori, we planned total and incremental cost and cost-effectiveness analyses from the provider perspective for cases of stunting averted, underweight, wasting, and infant mortality if a significant effect was observed at the 0·1 level. A detailed description of methods is presented elsewhere.^31^ Provider costs included costs to the implementing agencies and to public health-care providers. All costs were adjusted for inflation, discounted at 3% per year and converted to 2016 international dollars (INT$).

The statistician was masked to treatment when analysing the primary outcome for the final report presented to the DMC. Analyses were completed unmasked. This trial is registered as ISCRTN (51505201) and with the Clinical Trials Registry of India (number 2014/06/004664).

### Role of the funding source

Funders had no role in study design, data collection, analysis, interpretation, or writing of the report. The authors had full access to all data. NN and AP were responsible for the decision to submit for publication.

## Results

**Figure 2:**
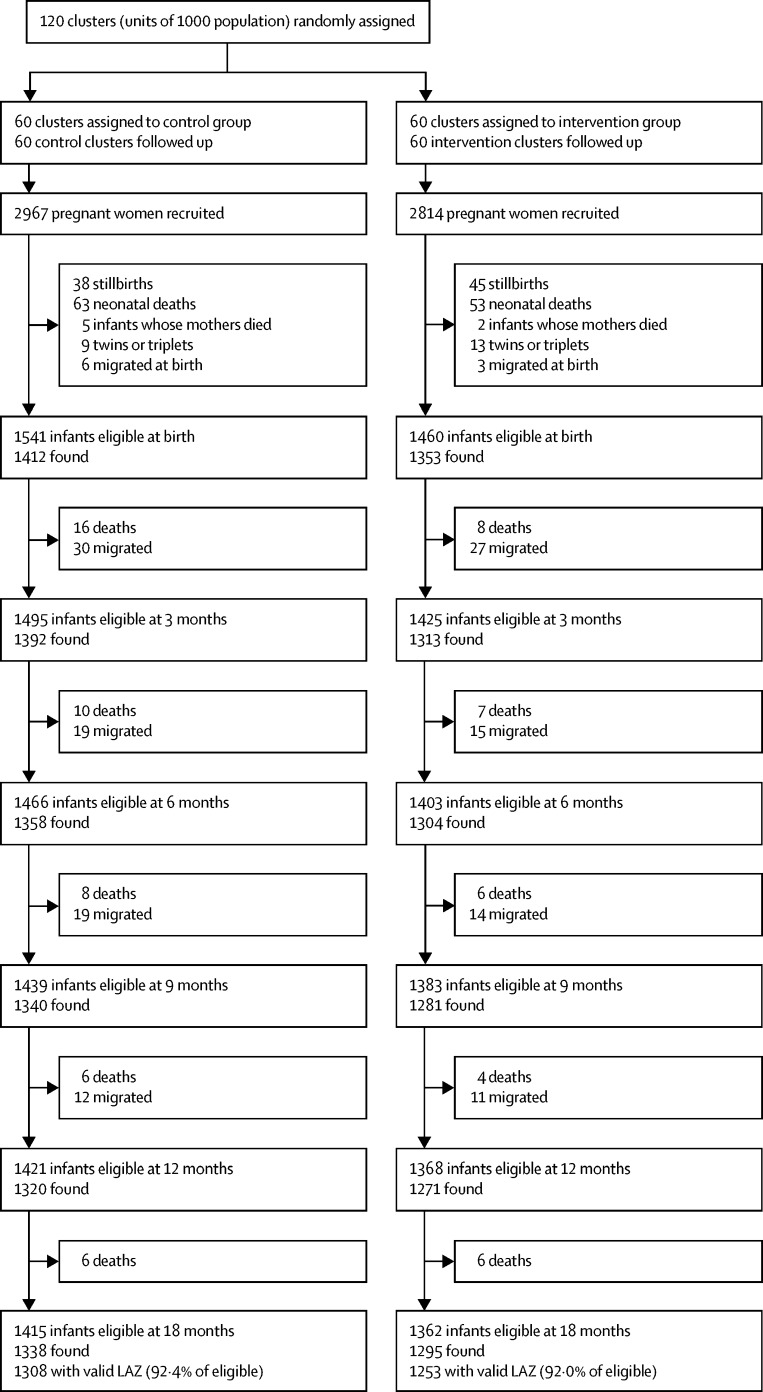
Trial profile LAZ=length-for-age *Z* score.

Between Oct 1, 2013, and Dec 31, 2015, we recruited 5781 pregnant women. 3001 infants were born to pregnant women recruited between Oct 1, 2013, and Feb 10, 2015, and were therefore eligible for follow-up (1460 assigned to intervention; 1541 assigned to control; figure 2). All clusters were retained in the study. Three groups of children could not be included in the final analysis for length-for-age *Z* score at 18 months: 147 migrated out of the study area (67 in intervention clusters; 80 in control clusters), 77 died after the neonatal period and before 18 months (31 in intervention clusters; 46 in control clusters), and seven had implausible length-for-age *Z* scores (<–5 SD; one in intervention cluster; six in control clusters). We had valid measurements for 1253 (92%) of 1362 eligible children at 18 months in intervention clusters and 1308 (92%) of 1415 in control clusters. We found no difference in tribal or caste status or wealth between children lost to follow-up and those retained in the trial at 18 months overall.

Socioeconomic characteristics of mothers whose children were eligible for follow-up were similar between intervention and control arms (table 1). In both arms, more than 75% of mothers were from scheduled tribe communities, and more than 50% could not read or only with difficulty. Mothers' mean age was also similar between arms (intervention mean 24 years, SD 4·6; control mean 23·9, SD 4·7). In both arms, less than 2% of households had access to a toilet.

**Table 1:** Characteristics of participants

		**Control**	**Intervention**	**All**
Mothers and infants eligible at birth		1541	1460	3001
District (state)				
	West Singhbhum (Jharkhand)	781 (51%)	727 (50%)	1508 (50%)
	Kendujhar (Odisha)	760 (49%)	733 (50%)	1493 (50%)
Residence				
	Main village	964 (63%)	889 (61%)	1853 (62%)
	Hamlet	577 (37%)	571 (39%)	1148 (38%))
Mother's age (years)		24·0 (4·6)	23·9 (4·7)	23·9 (4·6)
Parity		2·4 (1·6)	2·4 (1·6)	2·4 (1·6)
Class or caste status				
	Scheduled tribe	1199 (78%)	1100 (75%)	2299 (77%)
	Scheduled caste	71 (5%)	123 (8%)	194 (7%)
	Other backward class	269 (18%)	234 (16%)	503 (17%)
	Other	2 (<1%)	3 (<1%)	5 (<1%)
Land ownership				
	Less than 2 bighas	956 (62%)	837 (57%)	1793 (60%)
	Between 2 and 4 bighas	342 (22%)	355 (24%)	697 (23%)
	More than 4 bighas	110 (7%)	99 (7%)	209 (7%)
	Land mortgaged	6 (<1%)	7 (1%)	13 (<1%)
	No land	119 (8%)	145 (10%)	264 (9%)
	Missing	8 (1%)	17 (1%)	25 (1%)
Literacy				
	Can read easily	430 (28%)	418 (29%)	848 (28%)
	Cannot read or with difficulty	907 (59%)	874 (60%)	1781 (59%)
	Missing	204 (13%)	168 (11%)	372 (12%)
Education				
	None or less than 3 years	107 (7%)	119 (8%)	226 (8%)
	Primary	171 (11%)	201 (14%)	372 (12%)
	Lower secondary	283 (18%)	284 (19%)	567 (19%)
	Higher secondary and above	126 (8%)	124 (9%)	250 (8%)
	Missing	854 (55%)	732 (50%)	1586 (53%)
Multidimensional poverty quintiles				
	First (poorest) quintile	217 (14%)	221 (15%)	438 (15%)
	Second quartile	319 (21%)	286 (20%)	605 (20%)
	Third quartile	479 (31%)	463 (32%)	942 (31%)
	Fourth quartile	428 (28%)	410 (28%)	838 (28%)
	Fifth (richest) quintile	98 (6%)	80 (6%)	178 (6%)
Access to toilet				
	Yes—improved (flush or covered latrine)	14 (1%)	19 (1%)	33 (1%)
	Yes—not improved (open latrine)	9 (<1%)	10 (1%)	19 (1%)
	No toilet	1518 (99%)	1431 (98%)	2949 (98%)
Main source of drinking water				
	Tap	3 (<1%)	19 (1%)	22 (1%)
	Hand pump or tubewell	1027 (67%)	933 (64%)	1960 (65%)
	Covered dug well	24 (2%)	29 (2%)	53 (2%)
	Uncovered dug well	273 (18%)	273 (19%)	546 (18%)
	River, canal or spring	214 (14%)	206 (14%)	420 (14%)

Data are n (%) or mean (SD).

Between Oct 1, 2013, and Aug 10, 2015, 1293 mothers in the intervention arm were asked by data collectors if they had attended a group meeting or received a home visit in the past 3 months as part of their seven follow-up interviews. In 5068 (56%) of 9051 follow-ups, mothers said that they had attended a group meeting, and in 7241 (80%) they said that they had received a home visit. SPKs made an average of 37 home visits per month, four visits to pregnant women and 33 visits to mothers of children younger than 2 years, as compared with our anticipated 45–50 home visits. Home visits lasted a mean of 50 min each. Mothers received a mean of 11 home visits (SD 5) compared with the 19 visits planned. SPKs also supported 163 participatory groups (2–3 each), as planned. The five most common child-related problems prioritised by the groups were diarrhoea, which was prioritised by 100 (61%) of 163 groups, malaria (57%), worms (47%), low birthweight (36%), and acute respiratory infections (32%). The five most common maternal problems prioritised were food restrictions in pregnancy (61%), alcohol consumption in pregnancy (53%), closely spaced pregnancies (51%), anaemia (48%), and malaria in pregnancy (40%). The most common strategies addressed these problems through a combination of home-based preventive actions, care-seeking, and community-level activities such as planning kitchen gardens or campaigning (eg, against early marriage).

**Figure 3:**
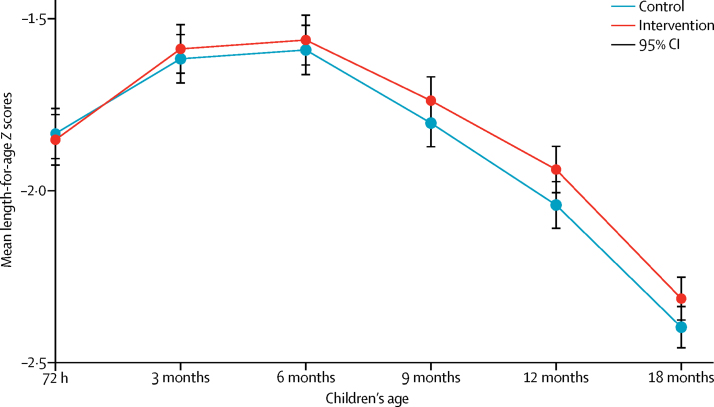
Mean length-for-age Z scores by allocation

Children's mean length-for-age *Z* score at 18 months was −2·31 (SD 1·12) in the intervention arm and −2·40 (SD 1·10) in the control arm (adjusted difference [aD] 0·107; 95% CI −0·011 to 0·226, p=0·08; figure 3, table 2). The intracluster correlation coefficient for length-for-age *Z* score at 18 months was 0·129 (95% CI 0·089–0·169). Children in intervention and control clusters had similar mean length-for-age *Z* scores from birth until 6 months, but these diverged gradually after 6 months (figure 3): the difference in mean length-for-age *Z* score (intervention – control) was −0·02 at birth, 0·03 at 3 months, 0·03 at 6 months, 0·06 at 9 months, 0·10 at 12 months, and 0·09 at 18 months (data not shown).

**Table 2:** Adjusted and unadjusted effects of intervention on children's and mothers' anthropometry

	**Control**	**Intervention**	Adjusted effect (95% CI)*	**p value**	**Unadjusted effect**†	**p value**
**Primary outcome**						
n	1308	1253	··	··	··	··
LAZ at 18 months	−2·40 (1·10)	−2·31 (1·12)	0·107 (−0·011 to 0·226)	0·08‡	0·104 (−0·034 to 0·242)	0·14
**Secondary outcomes (mother)**						
n	2952	2805	··	··	··	··
Maternal MUAC in third trimester of pregnancy	22·8 (1·81)	22·8 (1·89)	0·012 (−0·154 to 0·179)	0·88	0·021 (−0·167 to 0·208)	0·83
n	1240	1191	··	··	··	··
Maternal BMI at 9 months post partum	18·5 (2·13)	18·7 (2·24)	0·186 (−0·025 to 0·397)	0·08	0·190 (−0·021 to 0·402)	0·08
**Secondary outcomes (child)**						
n	1181	1167	··	··	··	··
Birthweight	2·56 (0·40)	2·57 (0·41)	0·012 (−0·022 to 0·047)	0·48	0·011 (−0·026 to 0·047)	0·57
n	1010	1014	··	··	··	··
Change in LAZ from birth to 18 months	−0·55 (1·27)	−0·48 (1·25)	0·074 (−0·062 to 0·211)	0·29	0·075 (−0·064 to 0·214)	0·29
n	1279	1231	··	··	··	··
WHZ at 18 months	−1·76 (1·04)	−1·76 (1·04)	0·019 (−0·075 to 0·114)	0·69	0·020 (−0·083 to 0·124)	0·70
n	1283	1236	··	··	··	··
WAZ at 18 months	−2·41 (1·05)	−2·37 (1·05)	0·068 (−0·023 to 0·159)	0·14	0·067 (−0·042 to 0·177)	0·23
n	1311	1261	··	··	··	··
MUAC at 18 months	13·3 (0·95)	13·4 (0·95)	0·050 (−0·072 to 0·173)	0·42	0·052 (−0·082 to 0·186)	0·44
n	1308	1253	··	··	··	··
Stunting (<2 SD LAZ) at 18 months	866 (66%)	807 (64%)	0·84 (0·66 to 1·08)	0·17	0·87 (0·663 to 1·135)	0·30
n	1279	1231	··	··	··	··
Wasting (<2 SD WHZ) at 18 months	533 (42%)	487 (40%)	0·88 (0·72 to 1·07)	0·22	0·88 (0·709 to 1·083)	0·22
n	1283	1236	··	··	··	··
Underweight (<2 SD WAZ) at 18 months	839 (65%)	772 (63%)	0·81 (0·66 to 0·99)	0·04	0·82 (0·654 to 1·038)	0·10

Data are mean (SD) or n (%). BMI=body-mass index. WHZ=. WHZ=weight-for-height Z score. WAZ=weight-for-age Z score. LAZ=length-for-age Z score. MUAC=mid-upper arm circumference.

* Adjusted effect is adjusted difference except for stunting, wasting, and underweight for which the adjusted effect is adjusted overall response. Adjusted for infant sex, district, multidimensional poverty index, residence (village or hamlet) and tribal status (all fixed effects) and cluster (random effect). All denominators include children and mothers available for follow-up and with valid anthropometric measurements using WHO 2006 Standards plausibility criteria for LAZ, WHZ, and WAZ.

† Unadjusted effect is difference except for stunting, wasting, and underweight for which the effect is overall response. Adjusted only for infant sex, district and cluster (random effect).

‡ Adjusted results with multiple imputation for missing data: adjusted difference: 0·100 (95% CI −0·028 to 0·227), p=0·13.

We detected no effect of the intervention on mean maternal mid-upper arm circumference in the third trimester of pregnancy (aD 0·012; 95% CI −0·154 to 0·179, p=0·88), or on maternal body-mass index (BMI) at 9 months post partum (aD 0·186; 95% CI −0·025 to 0·397, p=0·08). The odds of pregnant women reporting eating 3 times or more in the past 24 h were similar between intervention and control clusters (adjusted odds ratio [aOR] 1·14; 95% CI 0·78 to 1·67, p=0·49), but the odds of pregnant women reporting achieving minimum dietary diversity in intervention clusters were increased (aOR 1·40; 95% CI 1·03 to 1·90, p=0·0311).

Mean birthweight, weight-for-height *Z* score at 18 months, mid-upper arm circumference at 18 months, and the odds of children being stunted and wasted at 18 months were similar between intervention and control clusters. The odds of children being underweight at 18 months in intervention clusters were reduced (aOR 0·81; 95% CI 0·66–0·99, p=0·0436).

We found no difference between arms in the proportion of children who were exclusively breastfed until 6 months between intervention and control arms (intervention 51%; control 50%; aOR 1·06; 95% CI 0·74–1·50, p=0·75), or in the odds of children eating complementary foods by 6 months (intervention 50%; control 45%; aOR 1·30; 95% CI 0·78–2·13, p=0.30; table 3). The odds of children achieving minimum dietary diversity and meal frequency at 12 months and 18 months were increased in intervention clusters (minimum dietary diversity aOR 1·47; 95% CI 1·07–2·02, p=0·0016 and meal frequency 1·60; 1·04–2·47, p=0·0339).

**Table 3:** Adjusted and unadjusted odds ratios for intervention effects on other secondary outcomes

		**Control**	**Intervention**	Adjusted*		**Unadjusted**†	
				AOR (95% CI)*	p value	OR (95% CI)	p value
**Mothers' nutrition in pregnancy**							
Mothers eligible during pregnancy		2967	2814	··	··	··	··
	Ate ≥3 times in last day	2412 (81%)	2359 (84%)	1·14 (0·78–1·67)	0·50	1·14 (0·77–1·67)	0·49
	Minimum dietary diversity‡	958 (32%)	1042 (37%)	1·40 (1·03–1·90)	0·0311	1·40 (1·02–1·92)	0·04
**Infant and young child feeding**							
Infants eligible at 6 months		1368	1315	··	··	··	··
	Infants exclusively breastfed until 6 months	678 (50%)	673 (51%)	1·06 (0·74–1·50)	0·75	1·08 (0·76–1·53)	0·67
Infants eligible at 9 months		1439	1383	··	··	··	··
	Infants who started complementary foods at 6 months	650 (45%)	689 (50%)	1·30 (0·78–2·13)	0·30	1·28 (0·76–2·13)	0·34
Infants eligible at 12 months		1421	1368	··	··	··	··
	Children with minimum dietary diversity at 12 months	394 (28%)	515 (38%)	··	··	··	··
	Children given minimum meal frequency at 12 months	870 (66%)	936 (74%)	··	··	··	··
Infants eligible at 18 months		1415	1362	··	··	··	··
	Children with minimum dietary diversity at 18 months	627 (44%)	679 (50%)	··	··	··	··
	Children given minimum meal frequency at 18 months	1100 (82%)	1099 (85%)	··	··	··	··
	Children given a source of protein§ at 18 months	620 (44%)	679 (50%)	1·30 (1·00–1·69)	0·0486	1·30 (1·00–1·69)	0·05
Combined effect measure for 12 and 18 months together							
	Children with minimum dietary diversity	··	··	1·47 (1·07–2·02)	0·0161	1·47 (1·07–2·02)	0·02
	Children given minimum meal frequency	··	··	1·60 (1·04–2·47)	0·0339	1·59 (1·03–2·44)	0·04
**Morbidity and care for sick children**‡							
Infants eligible at 6 months		1338	1295	··	··	··	··
	Diarrhoea, cough, fever in past 2 weeks	508 (38%)	479 (37%)	0·90 (0·69–1·19)	0·47	0·92 (0·70–1·21)	0·57
Infants who experienced diarrhoea, cough, fever in past 2 weeks		287	322	··	··	··	··
	Received appropriate home care during illness episode¶	100 (35%)	114 (35%)	1·10 (0·64–1·90)	0·73	1·10 (0·63–1·94)	0·73
Infants who experienced diarrhoea, cough, fever in past 2 weeks		240	244	··	··	··	··
	Care sought from a nurse or doctor	43 (18%)	52 (21%)	0·87 (0·45–1·69)	0·69	0·93 (0·47–1·82)	0·84
**Infection control and hygiene**							
Infants eligible at 12 months		1421	1368	··	··	··	··
	Children received BCG, OPV3, DTP3, measles, hepatitis B vaccine	437 (31%)	441 (32%)	1·10 (0·74–1·64)	0·62	1·10 (0·72–1·67)	0·66
Infants eligible at 18 months		1298	1280	··	··	··	··
	Hands washed before feeding||	166 (13%)	438 (34%)	5·23 (2·61–10·5)	<0·0001	5·03 (2·53–10·0)	<0·001
	Hands washed after helping with defecation||	558 (43%)	919 (72%)	4·63 (2·98–7·20)	<0·0001	4·45 (2·88–6·86)	<0·001
	Hands washed after defecation||	579 (45%)	922 (72%)	4·40 (2·87–6·73)	<0·0001	4·18 (2·75–6·38)	<0·001
**Mortality**							
Infant deaths/livebirths (infant mortality per 1000 live births)		103/1613 (63·8)	78/1516 (51·4)	0·63 (0·39–1·00)	0·0496	0·60 (0·35–1·01)	0·06

Data are n (%) or mean (SD) unless otherwise specified. All secondary outcomes are self-reported. Deaths were verified by verbal autopsy. All denominators are eligible participants at each follow-up with data for the outcomes presented. DTP3=3 doses of diphtheria-tetanus-pertussis vaccine. OR=overall response. aOR=adjusted overall response. OPV3=3 doses of oral polio vaccine.

* Adjusted for infant sex, district, multidimensional poverty index, tribal status (all fixed effects), residence (village or hamlet) and village (random effect).

† Adjusted for district (fixed effects) and village (random effect) only.

‡ Defined as receiving foods from four or more food groups.

§ Includes animal or non-animal protein (eg, egg, soya bean chunks, or powdered grain with soya).

¶ Fluid replacement through oral rehydration therapy for diarrhoea and continued feeding during illness.

|| Hands washed by mothers.

The intervention had no effect on reported morbidity, care at home, or care-seeking for childhood illnesses: the odds of children having diarrhoea, cough, or fever in the past 2 weeks were similar in intervention and control clusters (intervention 37%; control 38%; aOR 0·90; 95% CI 0·69–1·19, p=0·47; table 3), as were the odds of receiving appropriate care for these symptoms at home (intervention 35%; control 35%; 1·10; 0·64–1·90, p=0·73), and the odds for care being sought from a qualified provider (intervention 21%; control 18%; 0·87; 0·45–1·69, p=0·69). The odds of mothers reporting washing their hands with soap before feeding children (5·23, 2·61–10·5, p<0·0001) and after helping a child with defecation (4·63, 2·98–7·20; p<0·0001) were increased in intervention clusters. Infant mortality was lower in intervention compared with control clusters (51·4 per 1000 livebirths *vs* 63·8 per 1000 livebirths, aOR 0·63, 95% CI 0·39–1·00, p=0·0496).

Increased exposure to group meetings and home visits was associated with higher length-for-age *Z* scores at 18 months, although none of the associations were significant (table 4). We found no differential effect of the intervention on length-for-age *Z* score at 18 months by sex (p value for interaction term 0·33), but a stronger effect in children belonging to richer quintiles of the multidimensional poverty index (p=0·0069; table 5).

**Table 4:** Effect on LAZ at 18 months by intensity of intervention exposure

	**n (%)**	**LAZ, mean (SD)**	Difference (95% CI)*	**Intensity p value**
**Exposure to women's groups across all follow-ups**†				
Control arm	1414 (51%)	−2·40 (1·10)	0	0·42 (categorical); 0·22 (linear)
None	196 (7%)	−2·31 (1·02)	0·058 (−0·123 to 0·240)	··
Attended <10 meetings	941 (34%)	−2·35 (1·14)	0·102 (−0·022 to 0·225)	··
Attended ≥10 meetings	219 (8%)	−2·17 (1·12)	0·173 (−0·002 to 0·348)	··
**Exposure to home visits across all follow-ups**†				
Control arm	1414 (51%)	−2·40 (1·11)	0	0·56 (categorical); 0·21 (linear)
Received <10 home visits	480 (17%)	−2·48 (1·09)	0·076 (−0·070 to 0·221)	··
Received ≥10 home visits	876 (32%)	−2·23 (1·13)	0·124 (−0·003 to 0·251)	··
**Exposure to group meetings and home visits across all follow-ups**†				
Control arm	1414 (51%)	−2·40 (1·11)	0	0·68 (categorical)
No meeting attended and <10 home visits	109 (4%)	−2·37 (1·03)	0·120 (−0·106 to 0·346)	··
Intermediate	1038 (38%)	−2·34 (1·13)	0·095 (−0·028 to 0·217)	··
≥10 group meetings and ≥10 home visits	209 (8%)	−2·17 (1·13)	0·164 (−0·014 to 0·342)	··

Subgroup analyses. MPI= multidimensional poverty index. LAZ=length-for-age Z score.

* Analyses adjusted for district, belonging to a scheduled tribe and scheduled caste, child sex, MPI, and whether hamlet or village residence (fixed effects) and for village (random effect).

† All exposures are self-reported.

**Table 5:** Intervention effect on LAZ at 18 months by infant sex and multidimensional poverty index quartiles

	**Control**		**Intervention**		Difference (95% CI)*	**Interaction p value**
	n	Mean LAZ (SD)	n	Mean LAZ (SD)		
**Infant sex**						
Female	732	−2·04 (1·10)	664	−1·90 (1·09)	0·145 (0·005 to 0·285)	0·33
Male	682	−2·78 (0·97)	692	−2·70 (1·01)	0·065 (−0·075 to 0·204)	··
**Multidimensional poverty index quintiles**						
First (poorest)	190	−2·77 (1·07)	197	−2·74 (1·08)	−0·033 (−0·255 to 0·189)	0·082 (categorical); 0·0069 (linear)
Second	292	−2·59 (1·03)	260	−2·53 (1·13)	0·020 (−0·190 to 0·229)	··
Third	440	−2·34 (1·14)	436	−2·27 (1·03)	0·145 (−0·026 to 0·315)	··
Fourth	398	−2·25 (1·09)	390	−2·12 (1·12)	0·207 (0·023 to 0·391)	··
Fifth (richest)	94	−1·96 (1·04)	73	−1·68 (1·18)	0·311 (−0·013 to 0·636)	··

LAZ=length-for-age Z score.

* Analyses adjusted for district, belonging to a scheduled tribe and scheduled caste, and whether hamlet or village residence (fixed effects) and for village (random effect).

The total annual cost of the intervention was INT$1 413 190 (INT$1 650 296 including village health sanitation and nutrition committee strengthening) and the mean cost was INT$423 957 (INT$495 089 including village health sanitation and nutrition committee strengthening). The mean annual cost of the intervention per livebirth was INT$290 and per pregnant woman covered was INT$151. If one considers beneficiaries to be all those living in the study areas, the average annual cost of the intervention was INT$7 per person covered. The incremental cost-effectiveness ratios were INT$29 561 per infant death averted (INT$34 520 including village health sanitation and nutrition committee strengthening) and INT$959 per life-year saved (INT$1120 including village health sanitation and nutrition committee strengthening; data not shown, the full cost analysis will be presented in a forthcoming publication).

## Discussion

In rural eastern India, the new community-based worker to promote interventions for growth during the first 1000 days of life, which we assessed in this trial, did not significantly increase children's length. The intervention did not have an effect on maternal and child anthropometric outcomes other than child underweight, and did not affect morbidity or care-seeking practices. The intervention did, however, improve self-reported dietary diversity and handwashing, as well as infant survival. The non-significant increments in length seen from 6 months of age onwards could, in part, be explained by increased dietary diversity among pregnant women and children, and improvements in hygiene through handwashing. Referrals for undernutrition were not among our secondary outcomes and will be reported in the process evaluation, but might have contributed to increased survival: referrals to Anganwadi workers for supplementary feeding or to malnutrition treatment centres doubled in the intervention area, due to the SPKs' referrals using mid-upper arm circumference.

The size of effect on length-for-age *Z* score seen in this study is in line with those reported in trials of nutrition education without supplementary feeding, and higher than those reported in previous trials of nutrition education and hygiene promotion globally and in India.^23^ A 2013 systematic review^13^ of five trials found that complementary feeding education led to a 0·23 (0·09–0·36) increase in height-for-age *Z* score in children younger than 2 years. In India, Bhandari and colleagues^20^ tested an intervention emphasising appropriate infant and young child feeding, handwashing, and adequate feeding during illness, but found no difference in attained length at 18 months. Another Indian trial by Vazir and colleagues^17^ found no significant differences in length at 15 months after an intervention to promote complementary and responsive feeding. These small or null effects on growth also echo results from the Alive & Thrive programme in Bangladesh, in which intensive infant and young child feeding counselling through home visits, community mobilisation, and mass media campaigns led to significant improvements in exclusive breastfeeding, timely initiation of complementary feeding, and consumption of protein, but no significant change in length-for-age *Z* score.^21^ The evidence available to date therefore confirms that community interventions to improve infant and young child feeding and hygiene through handwashing, care-giving and care-seeking during illness, though necessary, will only lead to small changes in length-for-age *Z* score.

This further underscores the importance of investing in girls and women's nutrition and going beyond immediate determinants to achieve substantial reductions in stunting. A 2016 analysis found that the leading risk factor for stunting among children younger than 2 years worldwide and in south Asia is being born small for gestational age (10·8 million out of 44·1 million cases), followed by poor sanitation (7·2 million cases) and diarrhoea (5·8 million cases).^32^ Dietary insufficiency in pregnancy, and groups' limited ability to influence the provision of clean water, sanitation, and key services in their villages could partly explain our small effect on length-for-age *Z* score.^33^ Ongoing trials are exploring the effects of combining nutrition-sensitive agriculture interventions and water, sanitation, and hygiene (WASH) interventions with nutrition-related behaviour change.^34^, ^35^ Even with considerable investment in health, nutrition, and WASH interventions, however, the reduction of stunting is perhaps best contemplated on a multigenerational horizon. In an analysis^36^ of longitudinal growth data from three Gambian villages where free primary and antenatal care, comprehensive immunisation, improved water and sanitation, and treatment for malnutrition were available, the prevalence of stunting among 2-year-olds halved between 1972 and 2012, but remained high at 30%. Countries with equity-oriented public policies, including income redistribution, universal access to education, health, water supply, and sanitation services, have achieved rapid and substantial declines in stunting, confirming the need for action on underlying and basic determinants of undernutrition.^37^

Our study had important strengths. We had a high follow-up rate and minimised technical error of measurement by carrying out three three standardisation exercises. Additionally, we traced children's growth at six timepoints, and the incremental gains in growth seen in the intervention arm compared with the control arm over time support our belief that this is a genuine effect. Our study also had limitations. First, several of our secondary outcomes, including dietary diversity and handwashing with soap, were measured using self-reported data. These data could be affected by social desirability bias, which might have led to over-reporting of desirable practices. However, the differences in practices between the two arms were not consistent for all self-reported outcomes, which suggests that bias is unlikely. Second, although both home visits and group meetings promoted age-appropriate stimulation for young children, we were unable to measure the effect of this on children's development due to difficulties in obtaining a validated and acceptable assessment tool for our multilingual, rural context. Finally, although the associations detected in our subgroup analyses are plausible, results should be interpreted with caution considering the number of tests performed.

The cost-effectiveness ratios of INT$959–1120 per life-year saved is well below India's gross domestic product (GDP) per capita of INT$6089 (in 2015). This indicates that the intervention is highly cost-effective in reducing infant deaths in this context, according to WHO criteria.^38^

Should a second worker be introduced in 200 Indian districts with a high burden of undernutrition? Our trial suggests this is feasible and could affect dietary diversity, handwashing, and infant survival, but a substantial effect would not be expected on maternal or child anthropometry in the short term. Let us consider alternative options to expand the coverage of nutrition-specific interventions for pregnant women and children younger than 2 years in rural areas. First, the working hours and honorarium of the existing Anganwadi worker could be increased to enable her to reach more pregnant women and children. This solution has proved unpopular with decisionmakers in the past because of concerns about the financial consequences of increasing honoraria for more than 14 million workers. Self-help groups are a promising platform through which to deliver health, hygiene, and nutrition interventions; however, they require trained facilitators, links to the health system, and no rigorous evidence currently exists about the effects of working with such groups on health and nutrition in the first 1000 days of life. The Mother's Absolute Affection programme, which was launched last year, mandates the training and incentivisation of accredited social health activists to promote infant and young child feeding in the first 2 years of life through home visits and group meetings, along with screening and referral for acute malnutrition with mid-upper arm circumference, activities very similar to those tested in this trial.^39^ The National Health Mission has also sanctioned the scale-up and incentivisation of accredited social health activists to conduct participatory learning and action meetings to improve maternal and child health across ten states. Future research might therefore explore how to optimise the home visiting and participatory women's groups materials created during this trial and other studies for accredited social health activists, so that the number and content of home visits aligns with those recommended in WHO's Caring for the Childs Healthy Growth and Development, and women's groups have an opportunity to discuss how to address the immediate and underlying determinants of child undernutrition in a voluntary, participatory, and context-appropriate manner.^40^ Such action will be necessary but not sufficient to achieve the ambitious goal of reducing stunting by 40% by 2025. This goal will require sustained, equity-focused investment in nutrition-sensitive interventions including clean water, sanitation, family planning, girls' education, and social safety nets.
