# Supplementary material for: Effect of participatory women's groups and counselling through home visits on children's linear growth in rural eastern India (CARING trial): a cluster-randomised controlled trial
Source: Lancet Glob Health. 2017 Sep 11;5(10):e1004–16. doi: 10.1016/S2214-109X(17)30339-X (PMC5640793; doi:10.1016/S2214-109X(17)30339-X)
Supplement: Supplementary appendix [file mmc1.pdf]

THE LANCET
Global Health

Supplementary appendix

This appendix formed part of the original submission and has been peer reviewed. We post it as supplied by the authors.

Supplement to: Nair N, Tripathy P, Sachdev HS, et al. Effect of participatory women's groups and counselling through home visits on children's linear growth in rural eastern India (CARING trial): a cluster-randomised controlled trial. *Lancet Glob Health* 2017; **5:** e1004–16.

SUPPLEMENTARY FILE

Supplementary Figure 1: Theory of change

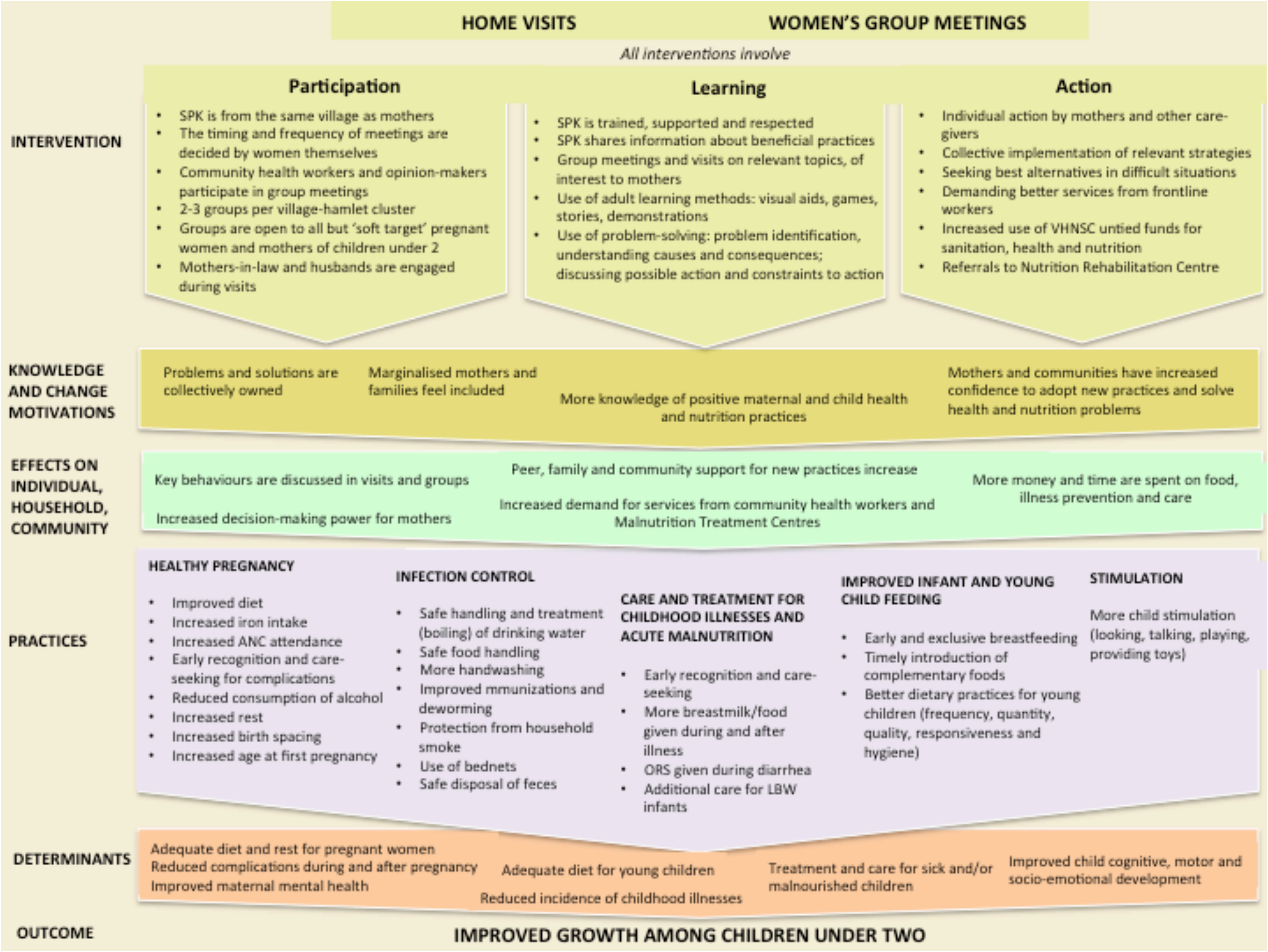

Supplementary Table 1: Counselling and referral activities during home visits

	**PREGNANCY**
**Antenatal care**	Ask the mother how many ANC check-ups; recommend at least four and discuss barriers to ANC
**Anaemia**	Look for symptoms of anaemia; refer to ASHA or ANM if present; if not advise a haemoglobin test as part of the next antenatal check up<br>Ask the mother if she is taking iron tablets and discuss where they are available, how many to take, and barriers to use
**Malaria**	Ask the mother about symptoms of malaria at any point in pregnancy and refer for a blood test if needed
**Diet and alcohol**	Ask the mother how many meals she is taking during the day; discuss the feasibility of increasing the number of meals<br>Ask the mother whether she is taking green leafy vegetables; discuss the feasibility of increasing by collecting locally available sources or making a kitchen garden<br>Ask the mother if she is taking alcohol; discuss the consequences of alcohol use in pregnancy (premature birth, growth and developmental problems)
**Birth preparedness**	Ask the mother if she has identified a skilled provider and made a plan for reaching the facility when labour starts, including plans for funding<br>Ask about signs of complications during pregnancy, labour and childbirth, and discuss these using picture cards<br>Ask if the family knows what community resources (emergency transport, funds) are available in case of an emergency and discuss locally available resources<br>Make a plan for emergencies (transport, companion and funds to reach a facility)<br>Encourage the mother and family to arrange supplies for birth (soft clean cloth, blanket, cloth for wrapping the child)
	**BIRTH TO SIX MONTHS**
**Referrals**	Ask the mother about her and the child's health<br>Support with referral to ASHA or ANM if the child is sick, and encourage breastfeeding
**Breastfeeding**	Ask the mother if she is breastfeeding her child.<br>Praise for breastfeeding. Ask how many times the child breastfeeds during the day and night<br>Breastfeed as often as the child wants, at least 8 times in 24 hours and when the child shows signs of hunger (fussing, sucking fingers, or moving lips)<br>Advise to encourage the child to empty the breast and then offer the other breast.<br>Ask about any other fluid given to the child besides breastmilk, and reasons for this<br>Say: "Only give breast milk. Breast milk alone will stop your child's thirst and hunger. Water can make them sick."<br>Ask about attachment and suckling; demonstrate positioning and attachment using picture and a demonstration<br>Ask about breastfeeding difficulties - encourage continued breastfeeding and refer to ANM for breast problems<br>Advise to breastfeed more if the child is recovering from illness
	**SIX TO 18 MONTHS**

**Referrals**	Ask the mother about her and the child's health<br>Support with referral to ASHA or ANM if the mother or child are sick, and encourage breastfeeding
**Breastfeeding**	Ask the mother if she is still breastfeeding her child.<br>If the mother still breastfeeds her child, praise her for continuing breastfeeding and encourage her to breastfeed as often as the child wants.<br>Ask the mother what fluids other than breastmilk she gives the child. Advise her to select nutrient – dense fluids (such as milk, soups) and NOT drinks with lower nutrient value (tea, sugary drinks & dilute sweet juices) that do not provide many nutrients.
**Complementary feeding**	Ask the mother what solid and semi-solid foods she normally gives to the child, and how often.<br><br>At 6 months, advise to initiate complementary feeding by offering the child small amounts of foods while continuing to breastfeed frequently.<br>Discuss mothers' concerns about initiation and help her resolve problems related to the availability of foods or conflicting family advice.<br><br>At 6–8 months of age, discuss offer 2–3 tablespoons (1 tablespoon = 15 ml) of thick porridge or well mashed foods 2–3 times per day.<br>Increase the amount gradually to 1/2 cup (1 cup = 250 ml). Discuss mothers' concerns about initiation and help her resolve problems related to the availability of foods or conflicting family advice.<br><br>By 8 months, give small chewable items to eat with fingers. Let the child try to feed by his/herself, but provide help. Offer 1–2 snacks between meals.<br><br>At 9–11 months of age, offer finely chopped or mashed foods and foods that child can pick up, about 1/2 cup, at 3–4 meals per day plus 1–2 snacks.<br>Feed the child's own plate or bowl. Discuss the feasibility of giving locally available animal source foods like poultry, fish and eggs as often as possible (ideally once a day).<br><br>By 12 months the child should have 3-4 meals per day plus 1-2 snacks in between meals.<br><br>If the quality of foods needs improving, ask the mother what types of food she is able to get and how often (prompt for foods available in the community: pulses, green leafy vegetables, mango/papaya/banana/grapes, meat, eggs, milk). On the basis of response discuss how to improve current recipes by adding oil/ghee, sugar (for density and taste) and vegetables/fruit/meat (for nutrients) and show new recipes. Plan at least four recipes and two snacks.<br><br>Ask the mother if she knows how to make instant feeds (e.g. satthu) and store it in an air-tight container. If not, demonstrate.
**Responsive feeding**	Encourage the mother and other caregivers to help the child to eat patiently and lovingly, actively encouraging, but not forcing. Demonstrate.<br><br>If the child loses interest while eating, encourage the mother and caregivers to remove any distractions and try to keep the child interested in the meal.<br><br>From 12 months, encourage the mother and other caregivers to feed the child from her own plate or bowl. Continue to actively help the child to eat. Talk to the child lovingly, look into her eyes and actively encourage her to eat, but do not force her.
**Measure MUAC**	Measure the child's MUAC and refer to AWW if MUAC <12.5cm
	**AT EACH VISIT TO A MOTHER AND CHILD:**
**Illness prevention**	Ask if the child often seems tired or sick such as with diarrhoea, cough or fever?<br>Ask what immunisations the child has received to date and refer to ANM if appropriate<br>Check the last time the child received a deworming tablet<br>Discuss likely causes of recurrent illness, especially diarrhoea, and make specific suggestions to reduce infections in her household.

	Suggest, discuss and problem-solve ways to maintain hygiene, which will help prevent infections:<br><br>Wash your hands with soap before preparing food, before feeding a child, and after using the latrine or toilet.<br>Obtain clean water for drinking, store it in clean covered containers, and use a clean scoop to draw it.<br>Wash your child's cup or bowl thoroughly with soap and clean water or boil it.<br>Cook foods thoroughly, especially meat, poultry, eggs and seafood. Reheat cooked food thoroughly; for example, bring stews to boiling point.<br>Cover food that is left over. Discard cooked food that is left at room temperature for longer than two hours.<br>Avoid contact between raw and cooked foods and store them in separate containers.<br>Collect the stool of a young child or child and put it into a latrine or bury it. Promptly clean a child who has passed stool, then wash your hands and the child's.
**Care for development**	Ask the mother how she thinks she child is developing.<br><br>Explain that brain development is rapid in the first two years of life, so young children need to be stimulated by playing with others, moving around, hearing sounds, and having things to see, touch, and explore. Play is children's work: the more they do, the more they learn and will be ready for school.<br><br>**Make age-specific recommendations for stimulation, and demonstrate them:**<br><br>**From birth to 6 months:** Provide ways for your child to see, hear, move arms and legs freely, and touch you.<br>Gently soothe, stroke and hold your child. Skin to skin is good.<br>Smile and laugh with your child. Talk to your child. Get a conversation going by copying your child's sounds or gestures.<br><br>**From 6-9 months:** Give your child clean, safe household things to handle, bang, and drop. Sample toys: containers with lids, metal pot and spoon. Respond to your child's sounds and interests. Call the child's name, and see your child respond.<br><br>**From 9-12 months:** Hide a child's favourite toy under a cloth or box. See if the child can find it. Play peek-a-boo. Tell your child the names of things and people. Show your child how to say things with hands, like "bye bye". Sample toy: doll with face.<br><br>**From 12-18 months:** Give your child things to stack up, and to put into containers and take out. Sample toys: Nesting and stacking objects, container and clothes clips. Ask your child simple questions. Respond to your child's attempts to talk. Show and talk about nature, pictures and things.

Supplementary Table 2: Plan of participatory learning and action meetings

	**Phase 1: Identifying problems**
1	Introduction to the meeting cycle (focus, number of meetings); Understanding social inequity, exclusion and discrimination; strengths of groups
2	Discussing the intergenerational cycle of undernutrition and current practices
3	Identifying & prioritizing problems related to maternal health and nutrition
4	Identifying & prioritizing problems related to child health and nutrition
	**Phase 2: Analysing problems and exploring solutions**
5	Understanding causes and solutions for the three prioritised problems (household level, community level and facility level)
6	Understanding causes and solutions for the three next prioritised problems (household level, community level and facility level)
7	Discussing and prioritizing possible strategies for implementing the solutions
8	Undertaking responsibilities, decide on the indicators and methods for measuring progress, and plan for community meeting
	Community meeting I
	**Phase 3: Taking action**
9	Discussion and Demonstration: asking members to bring locally available foods (including animal foods) - discussion about food diversity & what is given to children
10	Discussion and demonstration: *annaprasana* ceremony - timing of initiation of complementary feeding; demonstration of handwashing - enriching *kicheri*
11	Demonstration: making a nutritious powdered grain mix for children and discussing how to enrich it
12	Discussion and demonstration: safe handling of food and water
13	Prevention of childhood illnesses (card game: diarrhoea, ARI, worms)
14	Prevention of childhood illnesses (card game: malaria, measles)
15	Care and feeding during illness
16	Understanding the significance of a child's growth chart
17	Developing a Nutrition Garden
18	Understanding the importance of stimulation
19	Early childhood stimulation techniques
20	Supporting Early Childhood Development through everyday interactions
21	Childhood Malaria
22	Reinforcing caring and feeding practices for children aged 6-24 months
23	Care-seeking for Illnesses
24	Learn the beneficial practices discussed during home visit counselling sessions
25	Discussing possible strategies for improving maternal nutrition
26	Discussing contraception (focus on delaying first birth and spacing) - discussing temporary methods that are locally available (in presence of ANM and AWW) locally
27	Preventing early marriages and delaying first pregnancies
28	Understanding the pathways to good health and nutrition
	**Phase 3: Evaluation**
29	Evaluation of women's group activities by group members

Supplementary Table 3: Studies reviewed in the 'Research in context' panel

Main author	Mean unadjusted difference in LAZ (Intervention-Control)	Intervention, n	Intervention LAZ	Intervention SD	Control, n	Control LAZ	Control SD	Comparison
**LAZ**								
**Aboud 2011**	-0.18	85	-1.99	1.1	101	-1.81	1.1	Five group weekly sessions on responsive feeding and stimulation *vs.* routine government services
**Aboud 2013**	-0.03	111	-1.09	1.2	122	-1.06	1	Group meetings and home visits with messages about hygiene, responsive feeding, play, communication, discipline and nutrition foods *vs.* routine government services
**Menon 2016**	0.00	1099	-1.86	1.17	1100	-1.86	1.24	IYCF and handwashing promotion via intensified personal communication community mobilisation and mass media *vs* community mobilisation and mass media alone
**Nair 2017**	0.09	1253	-2.31	1.12	1308	-2.4	1.1	(1) Monthly home visits for IYCF, infection control, care-giving and stimulation from 3rd trimester of pregnancy until 24 months after birth, (2) Participatory women's groups meetings to address underlying determinants of maternal and child undernutrition (3) Strengthening of Village Health Sanitation and Nutrition Committees *vs.* Strengthening of Village Health Sanitation and Nutrition Committees alone
**Yousafzai 2014**	-0.10	701	-2.4	1.2	680	-2.3	1.1	Responsive stimulation added to routine health and nutrition counselling *vs* routine health, hygiene and nutrition education

Attained length	Difference in length (Intervention-Control)	Intervention, n	Intervention length (cm)	Intervention SD	Control, n	Control length (cm)	Control SD	Notes
**Bhandari 2003**	0.20	435	75.7	3.38	394	75.5	3.17	Monthly home visits IYCF and handwashing promotion

								and care-seeking promotion through home visits and group meetings
**Vazir 2013**	0.50	153	74.4	2.51	182	73.9	2.76	11 IYCF messages through home visits twice or four times a month (depending on age) + 8 messages on responsive feeding + 8 messages on developmental stimulation and five simple toys *vs control*

Supplementary Table 4: Risk assessment for studies reviewed in the 'Research in context' panel

	Random sequence generation (selection bias)	Allocation concealment (selection bias)	Blinding of participants and researchers (performance bias)	Incomplete outcome data (attrition bias)	Selective reporting (reporting bias)	Other bias
**Aboud et al. 2011**	Unclear risk: no description of random sequence generation	Unclear risk: no description of allocation method	Low risk: "Ten research assistants who were kept unaware of group assignment throught the study visited mothers and recruited them."	Low risk: attrition <10%	Unclear risk: all outcomes reported but no published protocol	Baseline imbalances were detected after randomisation
**Aboud et al. 2013**	High risk: Four unions where Save the Children Worked selected as sites; two randomly assigned to intervention and two to control	Unclear risk: no description of allocation method	Low: Research assistants were not aware of allocation and division between research and implementation team maintained	Low risk: attrition <10%	Unclear risk: all outcomes reported but no published protocol	None detected
**Bhandari et al. 2003**	Low risk: "right communities were paired on the basis of socioeconomic scores, child mortality and the prevalence of stunting and wasting, then pairs of communities were listed in alphabetical order. A statistician not involved with the study generated 4 single-digit random numbers using a random number table and allocated a community in a pair to the intervention group if the number was 0-4 and the second if it was 5-9.	Low risk: statistician was unaware of allocation	Unclear risk: mothers visited at home by workers not involved in intervention delivery. Blinding of research assistants to allocation not specified.	Unclear risk: Attrition was 15% and data on characteristics of children who dropped out not compared to those retained	Unclear risk: all outcomes reported but no published protocol	None detected
**Menon et al.**	Low risk: '100 districts across 5/6 divisions selected bt BRAC, narrowed to 78, then 4 sub-districts randomly selected for inclusion in evaluation sample, and 20 sub-districts randomised to one of two arms."	Randomisation carried out through a computer program in presence of program evaluators.	Unclear risk: 'households were not made explicitely aware of allocation; no blinding of allocation at the level of service delivery.	Low risk: baseline and endline; all clusters retained	Unclear risk: all outcomes reported but no published protocol	None detected

**Vazir et al.**	Low risk: 60 villages selected purposively from Integrated Child Development Services Areas and sets of three villages matched on maternal literacy, population size and birth rate.	Low risk: A researcher not familiar with the area used a random number generator to allocate villages to the three arms.	Low risk: assessment teams blinded to allocation and no interaction with intervention workers.	High risk: Attrition was 15% and data on characteristics of children who dropped out not compared to those retained; the most intensive intervention arm had the highest attrition (152/200 participants retained)	Unclear risk: all outcomes reported but no published protocol	None detected
**Yousafzai et al. 2014**	High risk: two sub-districts (Talukas) purposively selected, followed by two-stage stratified sampling to identify 20 Lady Health Worker catchment areas in each of the four arms.	High risk: random selection done independently from study etean for two arms out of four.	Low risk: Data collection team were masked to the intervention.	Low risk: attrition was 7%, though differing slightly by arm	Unclear risk: all outcomes reported but no published protocol	This trial is 2X2X2X2 factorial trial.
